# Comprehensive Numerical Simulation of Filling and Solidification of Steel Ingots

**DOI:** 10.3390/ma9090769

**Published:** 2016-09-09

**Authors:** Annalisa Pola, Marcello Gelfi, Giovina Marina La Vecchia

**Affiliations:** Department of Mechanical and Industrial Engineering, University of Brescia, via Branze 38, Brescia 25123, Italy; marcello.gelfi@unibs.it (M.G.); marina.lavecchia@unibs.it (G.M.L.V.)

**Keywords:** steel ingot, filling simulation, solidification, defects formation

## Abstract

In this paper, a complete three-dimensional numerical model of mold filling and solidification of steel ingots is presented. The risk of powder entrapment and defects formation during filling is analyzed in detail, demonstrating the importance of using a comprehensive geometry, with trumpet and runner, compared to conventional simplified models. By using a case study, it was shown that the simplified model significantly underestimates the defects sources, reducing the utility of simulations in supporting mold and process design. An experimental test was also performed on an instrumented mold and the measurements were compared to the calculation results. The good agreement between calculation and trial allowed validating the simulation.

## 1. Introduction

Notwithstanding the percentage of steel semi-products obtained via ingot casting is decreased during the last years, this method is still fundamental for specific low-alloy steel grades and for special forging applications, where components of large size are needed, such as mill rolls, turbine rotors, shafts for power plants, etc. [1,2,3,4]. Nowadays, the production of crude steel in the world reaches about 60 million tons [5].

Mold filling of heavy ingot can be performed in two ways: top pouring and bottom pouring, also called uphill teeming. In the top pouring, the molten stream is more exposed to air, suffering from reoxidation problems. As the pouring stream impinges on the melt surface inside the mold, it carries reoxidation products and mold powder, floating on it, back into the bulk, resulting in macro-inclusions. Moreover, during filling, metal splash adheres to the mold walls and produces surface defects on the ingot skin, which subsequently needs surface conditioning [6]. This makes the top pouring method not suitable for high-quality steels, which prefer bottom pouring [7,8]. In bottom pouring, the liquid steel flows from the ladle down to the trumpet and, passing through the horizontal refractory runner, it enters the nozzle or ingate upwards, reducing the exposure to air, the entrapment of mold powder and the occurrence of splashing. The bottom pouring needs a controlled velocity during filling in order to avoid turbulences and, consequently, powder entrapment or reoxidation defects [7]. 

The mold shape (round, square or multi-fluted cross section) also contributes to the casting quality. It is chosen according to the expected quality grade and, above all, to the product shape to be forged. Hence, in order to obtain sound ingots, mold shape, runners’ cross-section and length, as well as nozzle diameter and height have to be properly designed. Typically, the design is the result of the factory know-how but, in last decades, numerical simulation has been progressively applied as a useful tool for the optimization of mold shape and process parameters, to further improve the ingot quality. 

Different authors focused their investigations preferentially on the solidification phase of steel ingots production, mainly to predict defects formation [9]. Other authors, studying the solidification phase, also took into account the fluid field due to thermal buoyancy or melt superheat [10,11,12,13].

A more limited number of papers deal with the filling phase. Due to the more time consuming calculations, typically, the used geometrical models are simplified. In fact, neither the column nor the horizontal runner is normally considered, and a boundary condition of velocity at the whole cross section of the bottom inlet is conventionally fixed. Kermanpur et al. [14], for example, showed the effects of casting parameters, including the pouring rate, but focusing the attention on the solidification of the steel ingot. They found that pouring the melt under a constant rate in a mold with a low slenderness ratio and using a proper design for the hot top improve the casting quality. Tkadlečková et al. [15] investigated the effect of total filling time on porosity formation, showing that a decrease of the casting temperature together with longer filling time reduce the porosity level. Tkadlečková et al. [16] also showed a comparison between simulation and real casting. Zhang et al. [17], by using a simulation, determined two dimensionless factors able to evaluate the effect of casting parameters on the shrinkage porosity.

Some authors evaluated the effect of the fluid flow characteristics as a function of the entrance nozzle, like Eriksson et al. [18] who determined, for their case study, that a reduced risk of slag entrapment can be obtained using a 25 degree angle of the inlet nozzle. Marx et al. [19] assessed the influence of the feeding system design and the filling rate on ingot quality, also implementing user defined functions for agglomeration and trapping of inclusions at the free surface.

All these simplifications, even though valid, do not allow properly considering the real flow of the metal entering the mold. Aside by the teeming rate, in fact, the flow is strongly influenced also by the height of the column, the geometry of the horizontal runner and the entrance nozzle. To the authors’ knowledge, there is only one paper taking into account the whole geometry including trumpet, runner channel and mold. In that paper, Tan et al. [20] made a first attempt of comparing the results of a complete and reduced geometry simulation, but they focused their attention only on the very early stage of the filling and without considering the heat transfer and solidification.

The aim of this paper is to show the importance of simulating the complete mold geometry to appropriately predict the risk of defects formation, especially those due to non-metallic inclusions. As stated by Zigalo [21], in fact, during mold filling by bottom pouring technique inclusions can be formed because of the entrapment of mold flux. In particular, the emphasis of this analysis is not only on the initial stage but also on the entire mold filling, taking into account the thermodynamic aspects. 

To validate the numerical model, the results of the simulations were compared with the data collected during an experimental test performed on the industrial mold properly instrumented.

## 2. Experimental Procedure

The ingot casting test was carried out on a cylindrical four-mold system for the production of 19 ton round section ingots.

The mold consists of four parts made in hematite cast iron: the upper and bottom plates, the column and the ingot mold. The height of the ingot mold with the hot top (riser) is 5 m and the diameter is 840 mm; the height of the column is 6 m.

An insulating ring, 33 mm thick and 350 mm high, was placed at the top of each mold.

Pouring basin (trumpet) and running system were built up by using refractory bricks connected together. The diameter of the trumpet is 90 mm and the diameter of runners is 60 mm. The diverging entrance nozzle has a length of 260 mm and a diameter increasing from 60 to 80 mm. 

Calcium carbonate was used to fill the gap between the refractory bricks and the column and the plates.

Bags of mold powder were located about 80 mm from the base of the mold. When the molten steel reached the riser, additional isolating powder was distributed on the metal free surface. 

42CrMo4 steel grade was poured at 1560 °C with a time dependent flow rate, as explained below.

**Figure 1:**
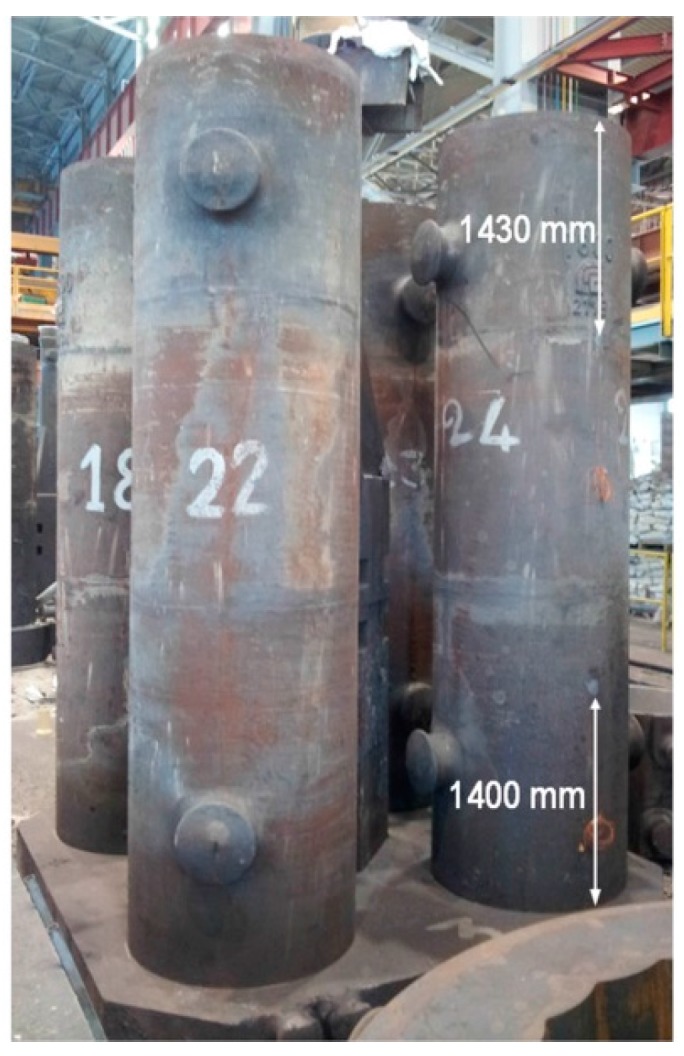
Four-mold system equipped with thermocouple.

Two K-type thermocouples, with a recording rate of 1 per 5 min, were placed on the surface of the cast iron mold: the first 1430 mm from the top and the second 1400 mm from the bottom, as shown in Figure 1. The temperature profile of the mold in these two points was recorded for 4 h.

The entire test, from the mold filling to the ingot stripping, was also monitored by means of a thermo-graphic camera Trotec IC120 (Trotec, Heinsberg, Germany), whose images where analyzed by IC-Report DuoVision Software (1.08.16S, Trotec). 

During the test, no other ingots were cast in the area around the four-mold system to avoid radiation effects, which can modify the temperature profile of the mold and ingots under investigation. However, self-radiation between column and molds as well as between the molds themselves is present.

## 3. Simulation Model

**Figure 2:**
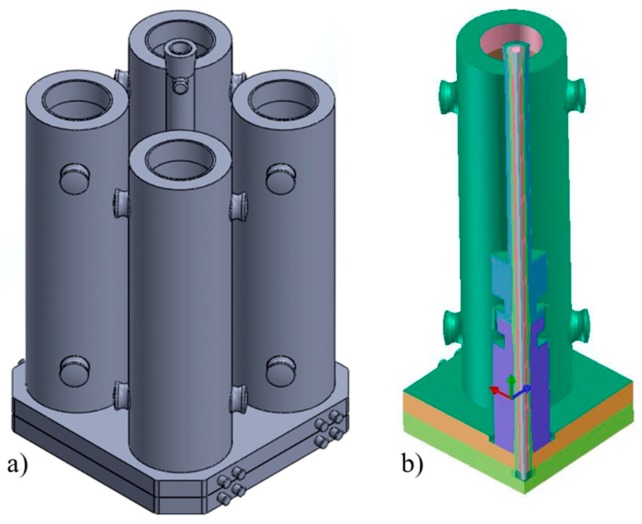
Geometry of the four-mold gating system (**a**); and used model (**b**).

A three dimensional model of the complete four-mold system was created, as shown in Figure 2a. 

Considering the double symmetry, to reduce the calculation time, only one mold was finally considered, applying a boundary condition of symmetry on two planes, as shown in Figure 2b.

The simulation of mold filling and casting solidification was performed by mean of the commercial software Procast^®^ (2015.0, ESI Group, Paris, France), based on finite element method (FEM). A mesh of 2,812,180 and tetrahedral elements for the comprehensive model was created. The software uses a FEM unstructured mesh with a full-layer option, which enables densification of nodes and tetra elements even if the surface mesh at this location has no interior nodes connecting the boundary edges. In addition, to increase the detail of flow solution, boundary layer option was applied. This is a very thin region of stagnated or even zero velocity flow that develops due to frictional effects with the wall. According to this accuracy in mesh definition, the results of this paper are mesh independent, as boundary layer elements allows handling wall stick-slip phenomena without any simplification for velocity conditions.

A simplified model, without the running system, was also created just eliminating the entire running system.

To calculate the fluid-dynamic field, the transport equations used in the simulation are the following:

- Energy equation
  (1) $$\rho \frac{\partial H}{\partial t}+\rho u_{i}\frac{\partial H}{\partial x_{i}}-\nabla \left[\left(k+\frac{\mu_{T}}{\sigma_{T}}\right)\nabla T\right]-q=0$$
  where ρ is the temperature dependent density, *H* is the temperature dependent enthalpy, *t* is the time, *u_i_* is the component of the velocity, *k* is the temperature dependent conductivity, µ*_T_* is the eddy viscosity, *q* is the spatial varying volumetric heat source, σ*_T_* Prandtl number, and *T* is the temperature.
- Continuity equation
  (2) $$\frac{\partial \rho }{\partial t}+\frac{\partial \rho_{i}}{x_{i}}=0$$
- Momentum equation
  (3) $$\rho \frac{\partial u_{i}}{\partial t}+\rho u_{j}\frac{\partial u_{i}}{\partial x_{j}}+\frac{\partial }{\partial x_{j}}\left[p\delta_{ij}\left(\mu +\mu_{T}\frac{\partial u_{i}}{\partial x_{j}}\right)\right]=\rho g_{i}-\frac{\mu }{K}u_{i}$$
  where *u_j_* is the component of the velocity, *p* is the pressure, δ*_ij_* is the Kronecker delta, µ is the viscosity, *g_i_* is the gravity acceleration, and *K* is the permeability.
- Turbulent kinetic energy equation:
  (4) $$\frac{\partial \left(\rho \bar{k}\right)}{\partial t}+u_{j}\frac{\partial \left(\rho \bar{k}\right)}{\partial x_{j}}-\frac{\partial }{\partial x_{j}}\left(\frac{\mu_{T}}{\sigma_{T}}\frac{\partial \bar{k}}{\partial x_{j}}\right)=\mu_{T}G-\rho \epsilon$$
  where $\bar{k}=\;1/2\left(u^{2}+v^{2}+w^{2}\right)$; *u*, *v* and *w* are the velocity components; ε is the turbulence dissipation rate; and $G=\left(\partial u_{i}/\partial x_{j}+\partial u_{j}/\partial x_{i}\right)\partial u_{i}/\partial x_{j}$ is the turbulence generation rate.

Concerning the mushy zone, it was modeled using the dimensionless Niyama approach reported by Carlson et al. [22]:

(5) $$N_{Y}^{*}=C_{\lambda }{\dot{T}}^{-1/3}N_{Y}\sqrt{\frac{\Delta P_{cr}}{\mu_{l}\beta \Delta T_{f}}}$$

where *C*_λ_ is the taken material constant, *Ṫ* is the cooling rate, *N_Y_* *= G*·*(Ṫ*)^0.5^ is the Niyama thermal parameter, *G* is temperature gradient, Δ*P* is the pressure drop across the mushy zone, µ*_l_* is the liquid dynamic viscosity, and β = (ρ_s_ − ρ_l_)/ρ_l_ is the total solidification shrinkage, function of the liquid and solid densities. 

Flow solution at free surface is solved by AMG solver (Algebraic Multi-Grid), especially modified with EBFEM solver (Edge-Based Finite Element Method) [23]. The thermal buoyancy effect is considered through the variation of steel density and solid fraction as a function of temperature, in the fluid-dynamics calculations.

**Figure 3:**
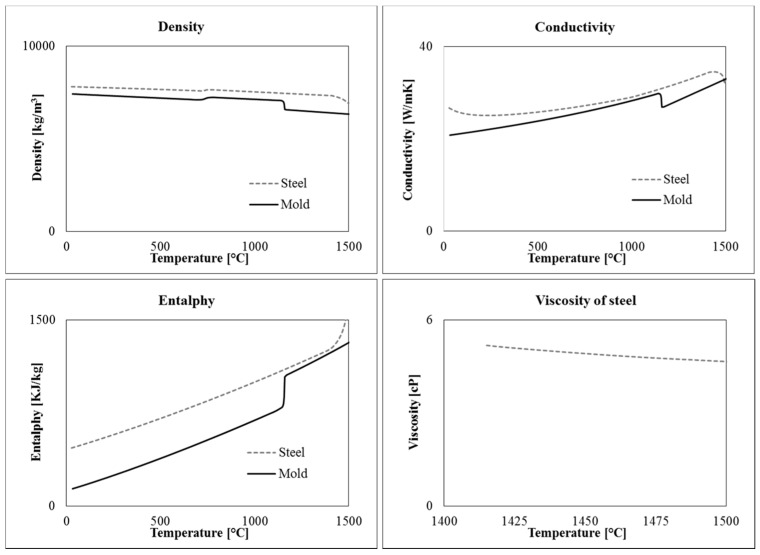
Thermophysical properties of mold and steel as a function of temperature.

To solve the equations, material data, initial and boundary conditions had to be properly defined. In particular, five different materials are present in the model: hematite cast iron, 42CrMo4 steel, refractory bricks, calcium carbonate and insulator. The temperature dependent thermophysical properties of steel and cast iron (conductivity, density, specific heat or enthalpy) are shown in Figure 3. They were calculated by means of Computherm Database^®^ (Pan Iron 5.0) available in Procast, as a function of the measured chemical composition and using the “Back Diffusion” model. The properties of insulating material and refractory were determined by the data supplied by the producers, which are in good agreement with those found in literature [12,13].

The steel was poured at 1560 °C. All the other materials were assumed to be initially at room temperature. External ambient pressure was also considered.

As known, the definition of the proper heat transfer coefficient at the interface between different domains is a crucial parameter to achieve reliable results in solidification calculation. The interface coefficient between mold and refractory/insulating materials was set at 100 W·m^−2^·K^−1^. The coefficient between molten steel and refractory/insulating materials was fixed at 200 W·m^−2^·K^−1^. The effect of gap formation at the steel/mold interface due to steel shrinkage was taken into account using a temperature dependent coefficient ranging between 150 and 500 W·m^−2^·K^−1^. All of the used coefficients were estimated in agreement with literature [9,14,24].

A boundary condition of convection with air was defined for the mold, considering the values proposed in [9]. Both the emissivity and the increase of air temperature around the mold with time, due to the solidifying steel, were taken into account. The radiation heat transfer was also assessed, considering the shadowing effect (or view factors), in order to calculate the interaction between the components (molds and molds and column) as well as with the environment. Hence, to include the environment into the model, an artificial “enclosure” was defined, which surrounds the mold and reproduces the effect of the environment.

The insulating effect of the mold powder, applied on the free surface of the incoming steel, was considered by reducing the heat exchange coefficient between steel and air to 3 W/m^2^/K and by reducing also the emissivity.

**Figure 4:**
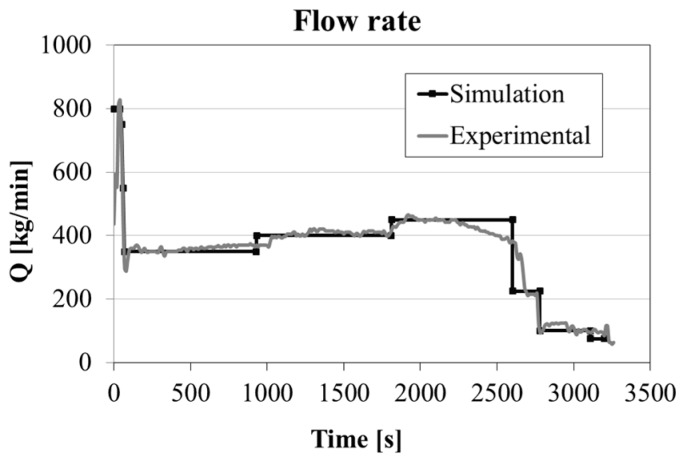
Teeming flow rate vs. time recorded during experiment and simulated.

As shown in Figure 4, a time dependent teeming flow rate was applied according to the procedure used in the steel plant for the experimental test. This boundary condition was applied at the top of the trumpet, considering the nozzle diameter of the ladle. The reported values of the flow rate applied at the trumpet are a quarter of the total amount, because of the used simplification of double symmetry.

## 4. Results and Discussion

**Figure 5:**
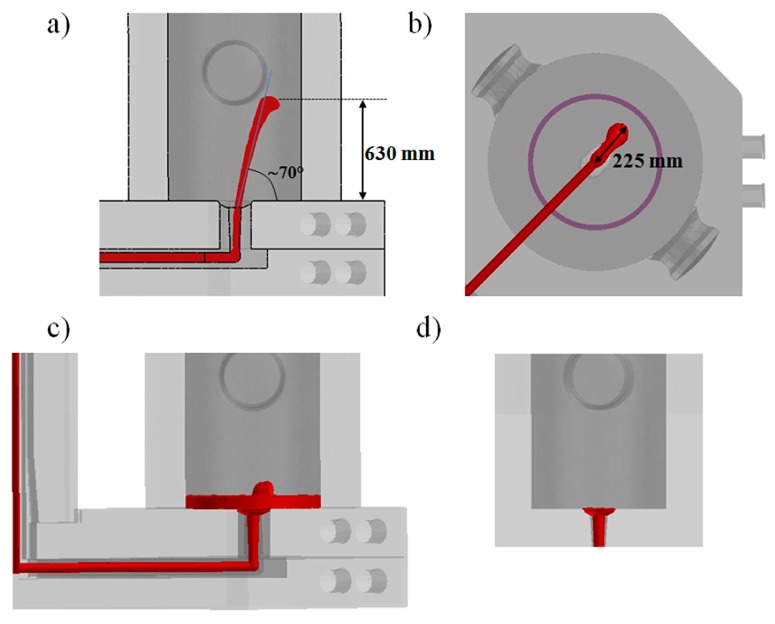
Entrance of steel spout in the case of complete mold with entire running system (**a**–**c**); and of simplified model (**d**).

As shown in Figure 5, during the first seconds the pouring phase is performed with a high flow rate to guarantee the complete opening of the nozzle and to avoid steel solidification in the refractory channels. Subsequently, in about 70 s, the flow rate is reduced down to 1400 kg/min. Because of this teeming rate, in the early stage of the process, the steel enters the mold at very high velocity, increasing the risk of air and powder entrapment [7]. In this condition, and without the presence of insulating seal or powder bags laid on the mold base, the steel spout reaches a height of about 630 mm before falling back under the gravity (Figure 5a). It can be additionally seen that the metal spout is not centered but shifted of about 225 mm from the center and oppositely to the column (Figure 5b), as a consequence of the entrance nozzle geometry and of the abrupt variation of flow direction at the elbow, i.e., from the horizontal runner to the vertical nozzle, whose connection is not properly designed. 

The knowledge of these data allows defining the height where powder bags can be suspended to avoid premature release of the insulating powder and subsequent entrapment. In fact, if the powder is released when a small pool of liquid metal has not been already formed and the metal does not occupy completely the inlet cross section (like in Figure 5a), the molten steel can easily engulf the fluxes, creating defects in the ingot. After less than 30 s, a pool of liquid is formed inducing a dumping effect on the incoming steel. The free surface is characterized by a light fluctuating effect, as detectable in Figure 5c, which stops when the pool reaches a level of about 250 mm and an almost flat free surface is settled.

For comparison, in Figure 5d the result of the conventional simplified simulation is shown, i.e., where the velocity is imposed at the bottom section of the entrance nozzle to minimize the calculation time, neglecting the running system. It can be clearly observed that the nozzle cross section is completely filled by the liquid steel, which enters the mold with a mushroom shape and fills the base of the cavity without big spout. In this case, no risk of premature release of the powder and subsequent slag entrapment can be predicted.

**Figure 6:**
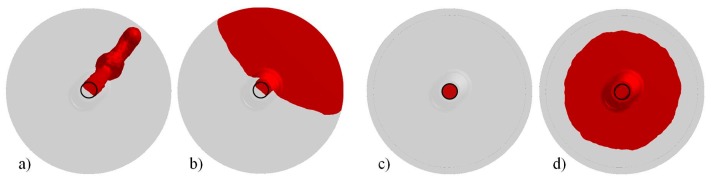
Bottom view of the steel entering the mold in case of: complete model (**a**,**b**); and simplified model (**c**,**d**).

In Figure 6, the bottom view of the liquid steel entering the mold from the nozzle is shown for both the comprehensive and simplified model. For the complete mold, it can be seen that the jet occupies less than 55% of the nozzle cross section, inducing an increase of velocity compared to the simplified case. The corresponding Reynolds number, calculated at the nozzle just before the exit, resulted in the order of 15,000 and 250,000 for the simplified and comprehensive models, respectively, demonstrating the presence of a turbulent flow, as stated in [25].

**Figure 7:**
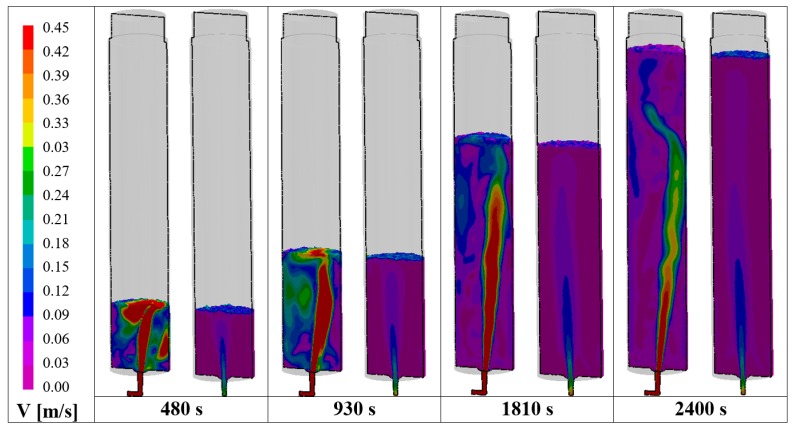
Filling sequence in terms of velocity distribution for both the: complete model (**left**); and simplified model (**right**).

Figure 7 reports the filling sequence in terms of velocity on the middle longitudinal section for both the comprehensive and the simplified models. In the case of the complete mold, the main stream remains shifted oppositely to the column during the entire filling, creating an asymmetric descending stream that can pull down the slag more easily, contrary to the case of the simplified simulation, where the main stream is almost centered. An eccentrically located flow can create a slag-free zone, the so-called eye, close to the wall, which can affect the detachment of slag particles from the main slag phase, as it happens in ladle stirring [26].

**Figure 8:**
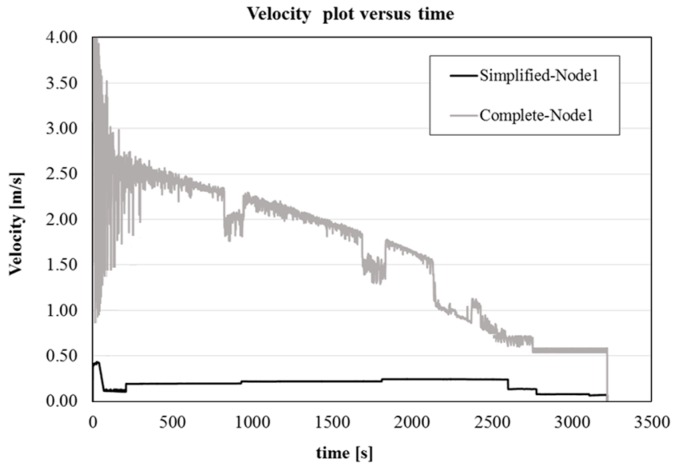
Comparison between the velocities recorded in the middle point of the nozzle exit for the simplified and comprehensive models.

Additionally, the complete model is characterized by a higher velocity of the jet, related to the not fully filled cross section of the runner, which induces also a higher velocity of the metal on the free surface, increasing the risk of powder entrapment. This can be better seen in Figure 8, where a plot of the velocity at the central point of the nozzle exit as a function of time is shown for both the simplified and complete simulation. For the comprehensive model, the velocity results higher and with a larger fluctuation, due to the stronger turbulence.

To reduce the risk of powder entrapment it is fundamental to decrease the deformation of the free surface, i.e., the jet velocity. This result can be achieved by properly design the running system, in particular the nozzle angle. Different authors have already shown the advantages of using a divergent nozzle, also with the addition of a swirl [18,20,27,28]. In this case, the filling should be strongly improved by increasing the inlet nozzle angle and by adding a shock absorber at the end of the horizontal channel.

During mold filling, the powder covering the liquid steel inside the mold forms a slag layer that floats on top of the rising molten steel [7]. An excessive turbulence at the powder–metal interface can cause reoxidation and exogenous inclusions. To evaluate the tendency for powder entrapment, the Weber number was calculated according to the modified equation reported by Erikson et al. [18]:

(6) $$We=\;\frac{u_{steel}^{2}\cdot \rho_{steel}}{\sqrt{\gamma g\left(\rho_{steel}-\rho_{slag}\right)}}$$

where *u_steel_* is the tangential steel velocity on the free surface of the molten steel; ρ*_steel_* and ρ*_slag_* are the density of the steel and of the powder, respectively; *g* is the gravity; and γ is the slag-steel interfacial tension. According to the finding of Xiao et al. [29], no problems occur when Weber number is lower than 12.3.

**Figure 9:**
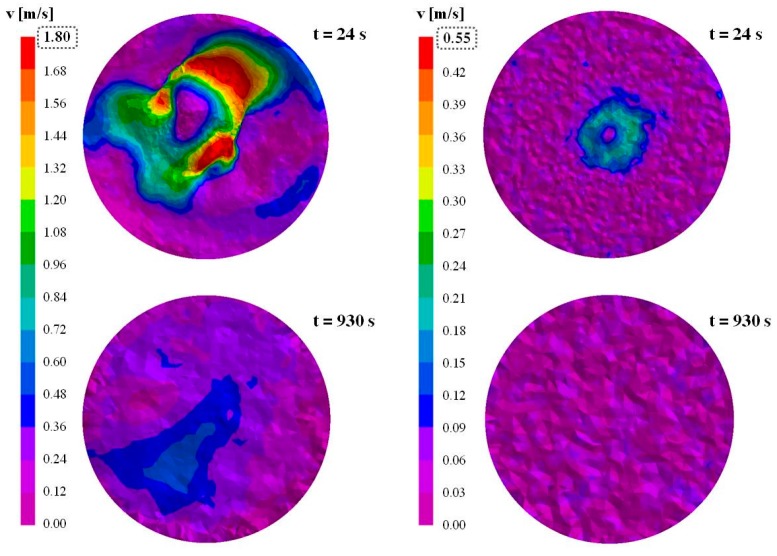
Tangential velocity at 24 and 930 s for the: complete model (**left**); and simplified model (**right**).

The tangential velocity of the liquid steel free surface was determined at 70, 930 and 1810 s, which correspond to the first variations of flow rate (Figure 3). Additionally, two intermediate times were also analyzed, i.e., 24 and 480 s, because high velocity values were observed during the first stage of mold filling. As an example, in Figure 9, the tangential velocity on the free surface at 24 and 930 s are reported, using different scales for the two models to better visualize the predicted values. It is evident that in the simplified model the velocity of the steel on the free surface results strongly lower than in the case of the complete simulation.

In Table 1, the maximum detected velocity in the above mentioned times are reported. 

For estimating the Weber number, the powder density was set at 2500 kg/m^3^, according to the literature data [18]. Four interfacial tension values were considered, in agreement with the ranges found in literature: 0.5 N/m [18], 1.0 N/m [30], 1.5 N/m [31] and 2 N/m [18].

**Figure 10:**
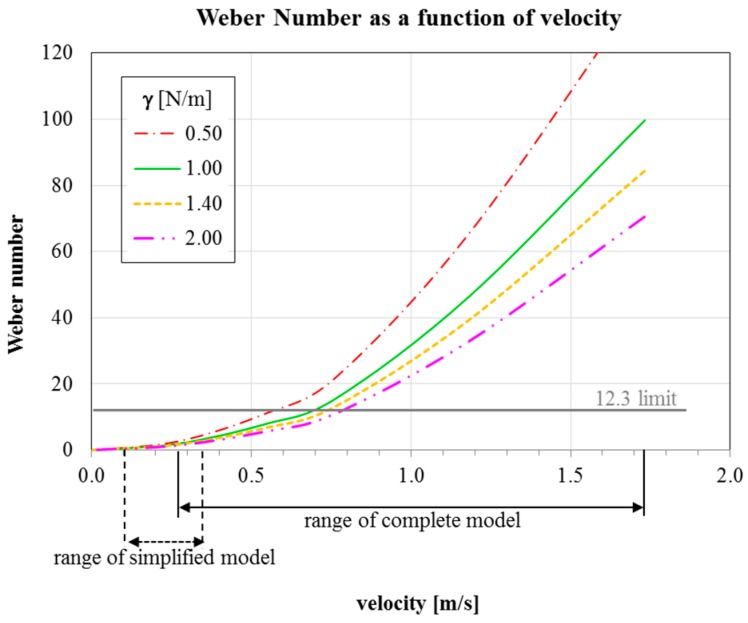
Variation of the Weber number as a function of the steel-powder interfacial tension.

In Figure 10, the determined Weber number as a function of the velocity between the steel and the slag for different interfacial tensions in shown. It can be seen that, in the case of the simplified model, the Weber number never exceeds the limit, independently from the considered velocity and interfacial tension, showing that no risk of powder entrapment can be expected. 

On the contrary, for the complete model, the Weber number is higher or very close to 12.3 for a wide range of velocities. Hence, the possibility of entrapping powder can be easily predicted. Considering the values in Table 1, it can be assessed that since 480 s the risk of defect formation is extremely high. 

At higher teeming time, when the mold is filled enough to reduce the velocity on the free surface, the risk is strongly reduced and it is disappears over 930 s, corresponding to about one third of the mold filling.

In the last steps, the flow rate is progressively reduced to 300 kg/min. Thus, considering the low velocity and the very high metallostatic head into the mold, the final phases of the filling are not dangerous for powder entrapment. 

Almost at the end of the filling, when the metal reaches the hot top, a covering powder is additionally applied to insulate the molten steel. This powder has a density of about 250 kg/m^3^ and, because of the low velocity of the free surface, no risk of entrapment seems to exist.

**Figure 11:**
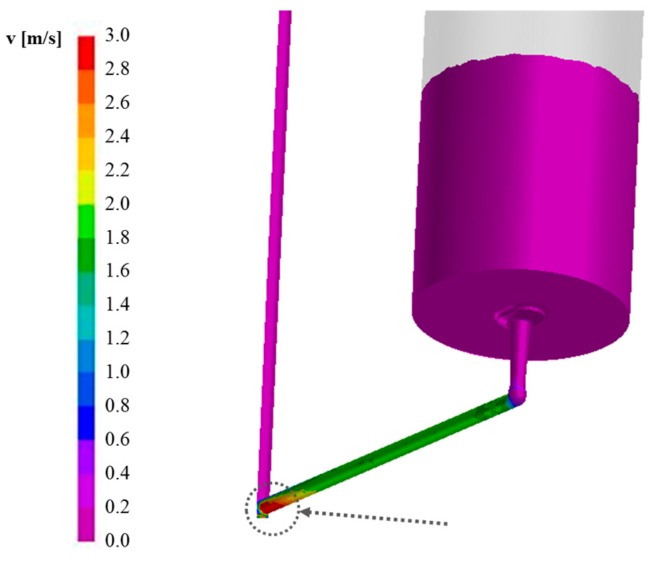
Shear velocity in the horizontal runner.

To avoid erosion and subsequent macro inclusions in the metal stream, the runners have to be designed in order to keep the shear velocity lower than 1 m/s, minimizing turbulences [7]. Considering the whole running system, it can be observed that the shear velocity of the molten steel flowing through the refractory channels is higher than this limit, particularly closed to the central distributor brick (Figure 11). This can allow the prediction of defects source and to determine optimal process parameters, contrary to the simplified model.

Finally, to verify the reliability of the developed simulation model, with complete runner system, they were compared the temperature profiles recorded by the thermocouples placed close to the top and close to the bottom of the mold (see Figure 1) and those resulting from the simulation as well as from the thermo-graphic camera. 

**Figure 12:**
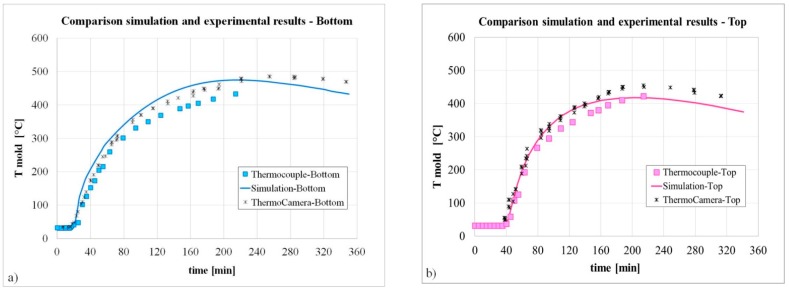
Comparison between simulation and experimental measurements for the bottom (**a**) and top (**b**) thermocouples.

As shown in Figure 12, a good agreement was obtained, demonstrating the reliability of the model. In particular, the increase in the mold temperature when the liquid steel reaches the level of the thermocouples, starting exchanging heat can be observed.

Slightly higher values of the measured temperatures than the calculated ones can be detected at during the ingot cooling (solidification is competed in around 3.5 h). This scatter at high temperature can be related to the limits of the measuring camera, in particular to the small variation of the emissivity, which was not set accordingly.

**Figure 13:**
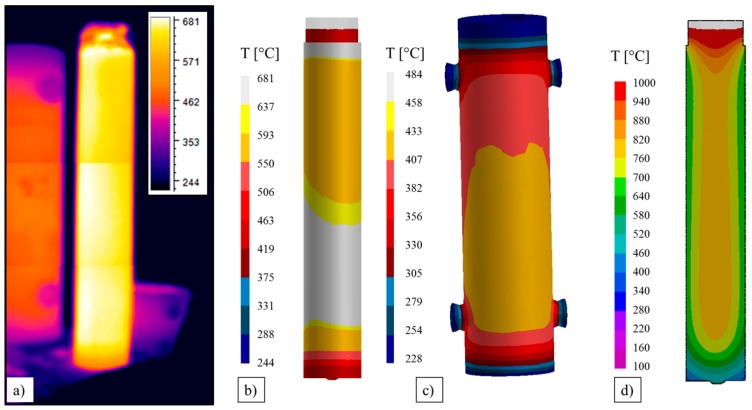
Temperature of the ingot on the surface just after the stripping phase: (**a**) measured by the thermo-camera; (**b**) simulated on the ingot; (**c**) simulated on the mold; and (**d**) temperature along the middle longitudinal section.

Just after the stripping, about six hours from the end of the filling, the measured temperature distribution on the ingot surface was compared to the predicted one. The good agreement between simulation and measurement (Figure 13a,b) allowed the further validation of the used model. The superficial temperature of the mold (Figure 13c) and also of the ingot resulted a little bit higher oppositely to the column and the adjacent mold, due to the radiation effects.

The temperature along the middle longitudinal section of the ingot (Figure 13d) shows an almost symmetric profile, only very limited moved towards the column, counterbalancing the effected induced by the shifted stream during the filling. 

**Figure 14:**
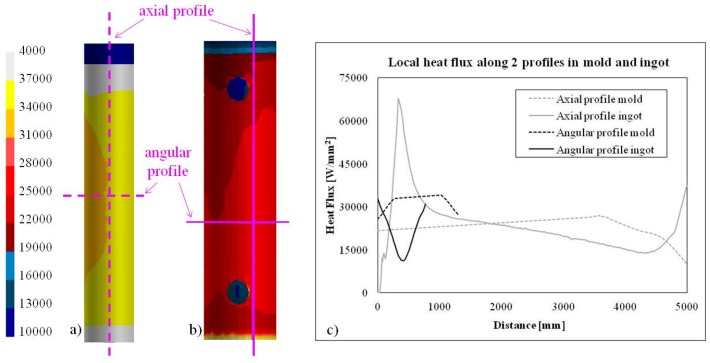
Heat flux along two profiles of the ingot (**a**) and mold (**b**); (**c**) Local heat flux as a function of the distance just after the stripping of the ingot.

In Figure 14, the heat flux on the surface of ingot and mold just after the stripping is reported, as well as its variation along an axial and angular profile. It can be seen that the heat flux resulted not axi-symmetrical mainly because of the radiation effect.

## 5. Conclusions

In this paper, the advantages of using complete instead of simplified models for the simulation of bottom-poured ingot casting was investigated. Starting from an industrial case, the not negligible effect of the running system on the prediction of mold filling and ingot quality was assessed. 

In particular, it was found that: in the early stage of the process, the steel spout is quite high and shifted oppositely to the column, as a consequence of the design of the running system (elbow and entrance nozzle geometry). This result can help in defining the position of the suspended bags to avoid premature release of the powder and subsequent entrapment. No similar prediction can be performed when the simplified model is used: the main stream remains shifted oppositely to the column also during the entire filling, creating a not symmetric descending stream able to drag the slag more easily, contrary to the case of the simplified simulation. A high risk of slag entrapment can be observed since the liquid steel reaches about one third of the mold, oppositely to the simplified model where the Weber number never exceeds the limit, hence establishing no risk of powder entrapment. Considering the whole running system, the shear velocity of the liquid steel along the refractory channel showed the risk of refractory erosion and, thus, of macro inclusions formation, particularly close to the central distributor brick. No remarkable differences in the temperature profiles during the ingot cooling were observed between the complete and the simplified models. These results determined the importance of simulating the complete mold when a deep analysis of defects sources is required.

Finally, the good fitting between calculated and recorded temperatures, both during filling and solidification as well as after the ingot stripping, allowed validating the simulation, demonstrating its reliability as predictive tool.

## Figures and Tables

**Table 1:** Steel velocity on the free surface as a function of time.

Time (s)	*v*_MAX_ for the Complete Model (m/s)	*v*_MAX_ for the Simplified Model (m/s)
24	1.73	0.35
70	1.18	0.32
480	0.76	0.25
930	0.55	0.20
1810	0.27	0.14
